# Protein Interactions in Genome Maintenance as Novel Antibacterial Targets

**DOI:** 10.1371/journal.pone.0058765

**Published:** 2013-03-11

**Authors:** Aimee H. Marceau, Douglas A. Bernstein, Brian W. Walsh, Walker Shapiro, Lyle A. Simmons, James L. Keck

**Affiliations:** 1 Department of Biomolecular Chemistry, University of Wisconsin School of Medicine and Public Health, Madison, Wisconsin, United States of America; 2 Department of Molecular, Cellular, and Developmental Biology, University of Michigan, Ann Arbor, Michigan, United States of America; Saint Louis University, United States of America

## Abstract

Antibacterial compounds typically act by directly inhibiting essential bacterial enzyme activities. Although this general mechanism of action has fueled traditional antibiotic discovery efforts for decades, new antibiotic development has not kept pace with the emergence of drug resistant bacterial strains. These limitations have severely restricted the therapeutic tools available for treating bacterial infections. Here we test an alternative antibacterial lead-compound identification strategy in which essential protein-protein interactions are targeted rather than enzymatic activities. Bacterial single-stranded DNA-binding proteins (SSBs) form conserved protein interaction “hubs” that are essential for recruiting many DNA replication, recombination, and repair proteins to SSB/DNA nucleoprotein substrates. Three small molecules that block SSB/protein interactions are shown to have antibacterial activity against diverse bacterial species. Consistent with a model in which the compounds target multiple SSB/protein interactions, treatment of *Bacillus subtilis* cultures with the compounds leads to rapid inhibition of DNA replication and recombination, and ultimately to cell death. The compounds also have unanticipated effects on protein synthesis that could be due to a previously unknown role for SSB/protein interactions in translation or to off-target effects. Our results highlight the potential of targeting protein-protein interactions, particularly those that mediate genome maintenance, as a powerful approach for identifying new antibacterial compounds.

## Introduction

The major public health threat posed by drug-resistant bacterial infections makes the development of new antibiotics a top biomedical priority [1]–[3]. Currently, four chemical scaffolds account for the vast majority of prescribed antibiotics and only nine direct molecular targets have been effectively exploited in bacteria. Adding to these limitations, many pharmaceutical companies have abandoned their antibacterial development efforts while those that have continued have found that traditional targeting of enzyme active sites yields fewer new drugs than earlier lead-discovery campaigns. These constraints have reduced the therapeutic approaches available to fight drug-resistant bacterial infections and highlight the need for new strategies to identify novel antibacterial lead compounds and molecular targets.

An emerging alternative lead-discovery approach investigated here takes advantage of small-molecule inhibitors that block essential protein-protein interactions (PPIs) as a new type of antibacterial agent. PPIs range in complexity from simple dimeric complexes formed between two proteins to intricate networks in which “hub” proteins bind simultaneously to many protein partners. PPIs are essential for nearly every cellular process and successes in developing therapeutic PPI inhibitors against eukaryotic targets (reviewed in [4]) suggest that such sites could be fruitful for antibacterial drug discovery. Indeed, compounds that disrupt protein complexes formed by the FtsZ bacterial cell division protein have been found to have antibacterial activity [5]–[8]. These observations support the idea that small molecules capable of blocking essential PPIs found uniquely in bacteria could provide novel broad-spectrum therapeutic tools to fight the growing number of drug resistant bacterial infections.

Bacterial single-stranded (ss) DNA-binding proteins (SSBs) are homotetrameric proteins that bind and protect ssDNA formed during cellular genome maintenance processes such as DNA replication and homologous recombination [9]. SSBs also function as organizational hub proteins by binding and recruiting over a dozen different genome maintenance enzymes to their cellular sites of action. The direct protein interactions are mediated by SSB's evolutionarily-conserved C-terminus (SSB-Ct: -Asp-Asp-Asp-Ile-Pro-Phe in *Escherichia coli* and -Asp-Asp-Asp-Leu-Pro-Phe in *Bacillus subtilis*) [10]. In *E. coli*, deletion or mutation of the SSB-Ct Phe is lethal, whereas altering the SSB-Ct Pro to Ser causes temperature-sensitive lethality [11]–[14], highlighting the essential role of SSB interactions. Structural studies of several proteins in complex with synthetic SSB-Ct peptides have shown that SSB-Ct binding sites share a common electrostatic arrangement that complements elecronegative groups from the Phe (α-carboxyl) and Asp (side chains) residues along with the hydrophobic Phe side chain [10], [15]–[19]. Although eukaryotic SSBs (Replication Protein A) also interact with a wide variety of protein partners [20], [21], they do so using mechanisms that are distinct from bacterial SSBs as they lack analogous SSB-Ct sequences.

**Figure 1:**
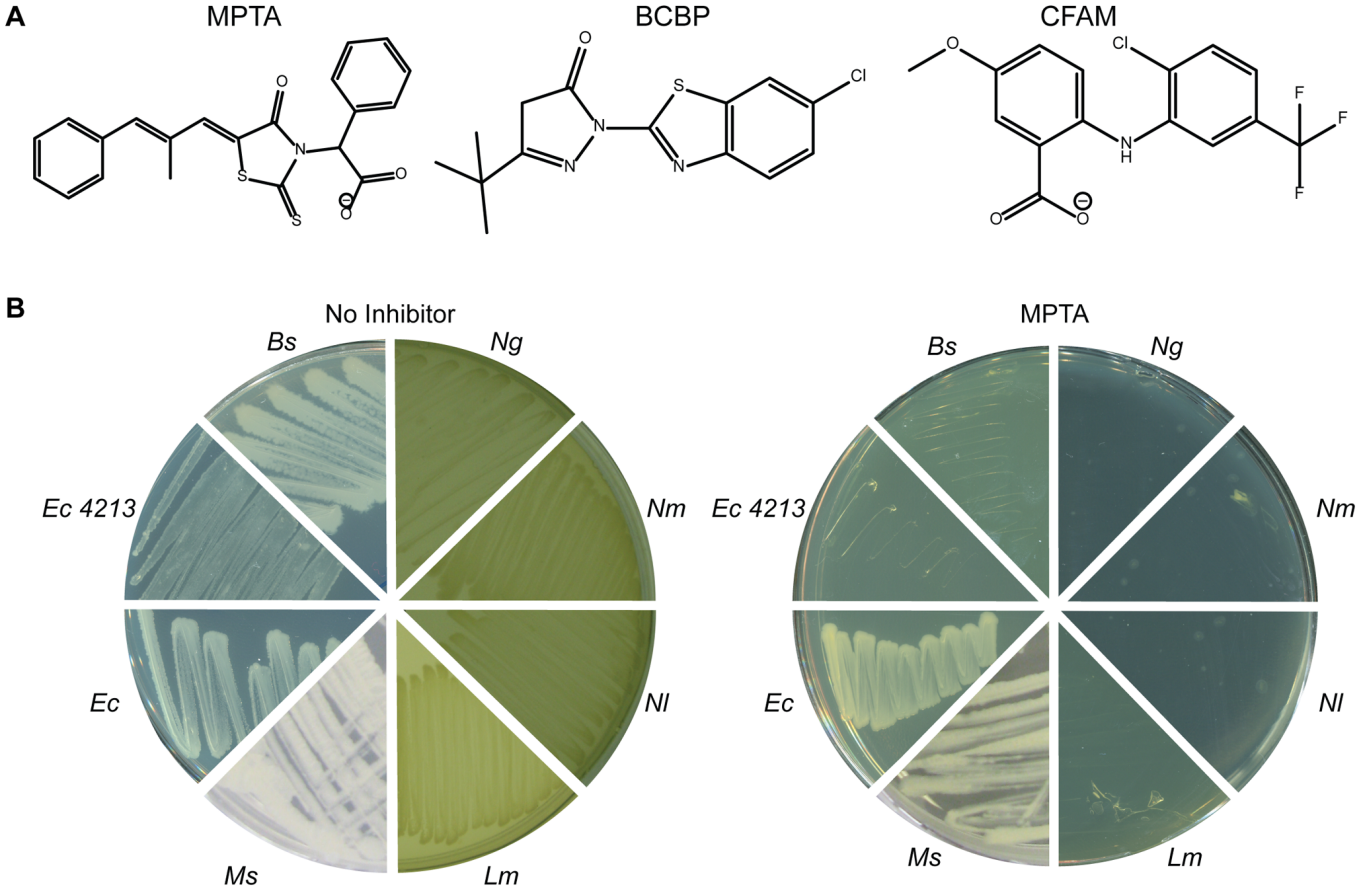
MPTA, CFAM and BCBP inhibit the growth of multiple prokaryotic species. **A.** Structures of MPTA, BCBP and CFAM [22]. **B.** Colony formation of several bacterial strains in the absence (left) or presence (right) of 50 µM MPTA. *E. coli* (*Ec*), *E. coli imp4213* (*Ec 4213*), *B. subtilis* (*Bs*), *Neisseria gonorrhoeae* (*Ng*), *Neisseria meningitidis* (*Nm*), *Neisseria lactamica* (*Nl*), *Listeria monocytogenes* (*Lm*), and *Mycobacterium smegmatis* (*Ms*) were plated without (left) or with MPTA (right). Identical experiments were performed with BCBP and CFAM, with the results summarized in Table 1.

Small molecules that disrupt *E. coli* SSB interaction with one of its binding partners (Exonuclease I) have been identified [22], [23] (Figure 1A). Of these PPI inhibitors, MPTA is a structural mimetic of the SSB-Ct Pro-Phe dipeptide and it broadly inhibits SSB/protein interactions, blocking complex formation with both Exonuclease I and additional binding partners (RecQ and PriA DNA helicases) [22]. In contrast, BCBP and CFAM are not obvious structural mimics of the SSB-Ct and each exhibits more specific inhibition of the SSB/Exonuclease I interface, with less potent activity against SSB/RecQ and SSB/PriA complexes. Structural and mechanistic studies further showed that, in spite of their structural differences, each of the inhibitors directly competes with the SSB-Ct for binding to Exonuclease I [22]. However, the effects of MPTA, BCBP, and CFAM on cellular genome maintenance reactions and their potential as antibacterial lead compounds have not been investigated.

Here, we test the hypothesis that SSB PPI inhibitors could serve as novel antibacterial agents. Each of the SSB PPI inhibitors is shown to have antibacterial activity against a diverse panel of bacterial species, with the more general SSB PPI inhibitor, MPTA, having the most potent activity. Treatment of *B. subtilis* with lethal doses of the compounds leads to rapid cessation of DNA replication and recombination, and ultimately to cell death. Similar results are observed with an *E. coli* strain that has heightened small-molecule membrane permeability properties. The inhibitors also unexpectedly inhibit protein synthesis, which could reflect inhibition of previously unknown roles for SSB/protein interactions in translation or to off-target activities of the compounds. Interestingly, evolved resistance to the compounds in the *E. coli* strain requires suppression of the hyperpermeability phenotype, which suggests that resistance mechanisms could be restricted to those that alter cytoplasmic availability of the SSB PPI inhibitors. Our results support a model in which the small molecules act by disrupting essential SSB/protein interactions in vivo and suggest that targeting essential PPIs that mediate genome maintenance could be a productive approach for developing novel antibacterial compounds.

## Materials and Methods

### Plate-based antibacterial sensitivity experiments

Plates of Luria broth [24], Middlebrook 7H10 (Fisher Scientific, DF0627-17-4), Mueller-Hinton (Sigma-Aldrich, 97580-500G-F), GC (Fisher [25]) and yeast peptone dextrose agar (YPD) were made with 50 µM MPTA (OTAVA #0105970015), 100 µM BCBP (Maybridge, #SEW01297) or 100 µM CFAM (Maybridge, #S07197). *E. coli* (strain MG1655) and *L. monocytogenes* (strain 10403S) were grown on LB agar. *B. subtilis* (strain PY79), *E. coli imp4213* and *S. aureus* (strain 33591) were grown on LB or Mueller Hinton agar. *N. gonorrhoeae* (strain MS11), *N. meningitidis* (strain 13102) and *N. lactamica* (strain 23970) were grown on GC agar. *M. smegmatis* (strain mc^2^155), *M. avium paratuberculosis* (strain K10), *M. bovis* (strain BCG) and *M. tuberculosis* (strain H37Rv) were grown on Middlebrook 7H10 agar. *S. cerevisiae* was grown on YPD agar. Strains were plated from a frozen stock or a liquid culture onto the appropriate medium and grown overnight (or for several weeks for the *Mycobacterial* strains).

### Minimum Inhibitory Concentration (MIC) measurements

10^6^ CFU/mL of *B. subtilis* or *E. coli imp4213* was inoculated into liquid media or onto solid plates with 1–100 µM of each compound in 3–5 independent experiments (method was adapted from the NCCLS [26]). Concentrations at which visible growth was terminated are reported as the MIC.

### Time-kill experiments

Triplicate cultures of *B. subtilis* PY79 (1×10^6^ CFU/ml) at 37°C were grown in media supplemented with 0.5×, 1×, 2×, or 4× the MIC of MPTA, BCBP or CFAM, or with DMSO (Fisher, BP231-1), 1 µg/mL mitomycin C (Sigma, M4287), or 25 µg/mL nalidixic acid (Fisher, AC16990-0050) as controls. The number of CFU/mL of culture was measured from aliquots (0, 1, 2, 4, 8, and 24 hours) that were serially diluted in sterile saline, plated on LB agar, and incubated at 37°C [27]. The lower limit of detection for colony counts was 100 CFU/mL. The time dependent killing curves were constructed by plotting mean colony counts over time; error bars represent one standard deviation of the mean.

**Figure 2:**
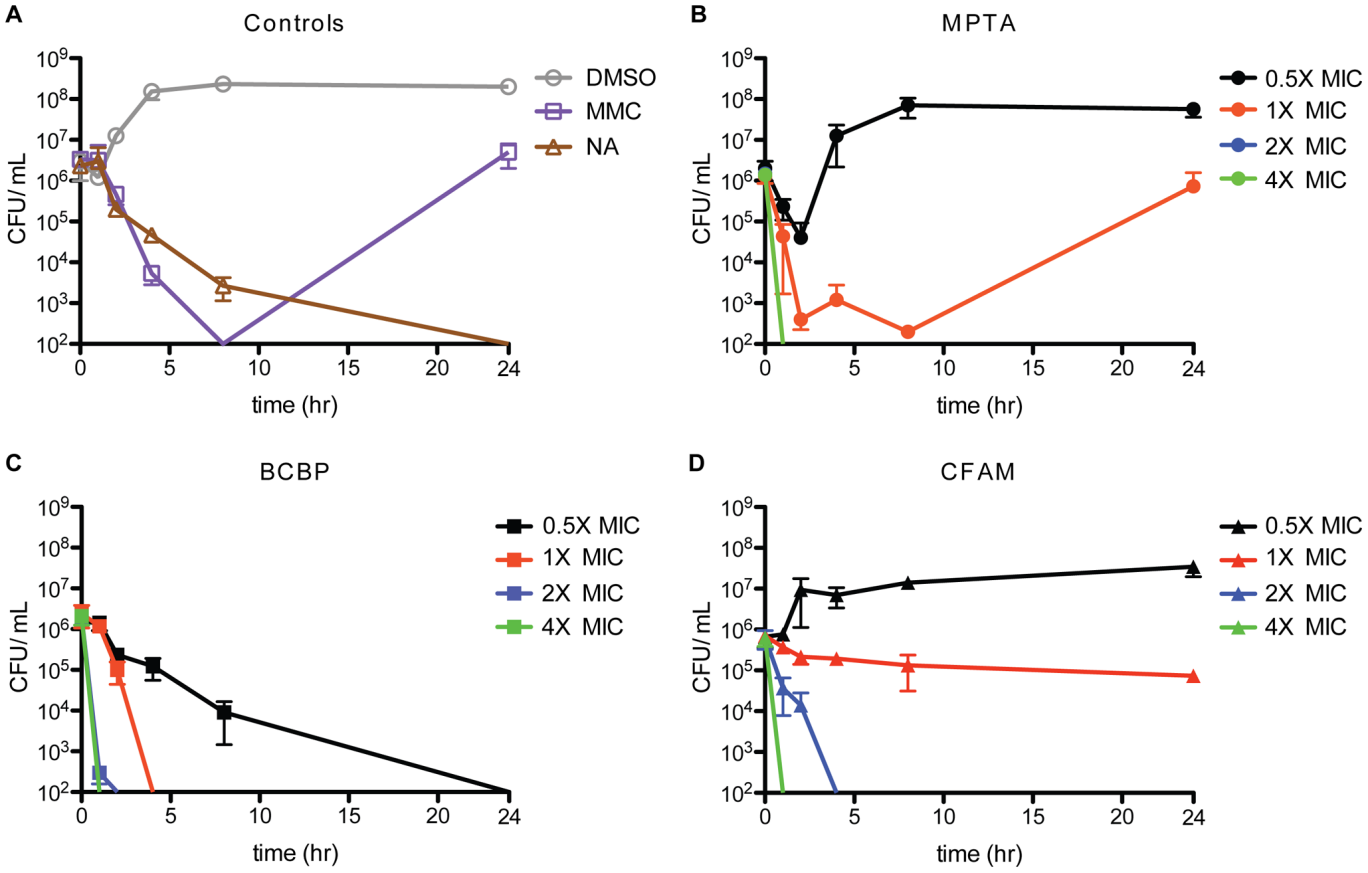
MPTA, BCBP, and CFAM are bactericidal. Time kill curves of *B. subtilis* PY79 treated with (**A**) controls (Dimethyl sulfoxide (DMSO), mitomycin C (MMC), nalidixic acid (NA)), (**B**) MPTA, (**C**) BCBP, or (**D**) CFAM at 0.5×, 1×, 2×, or 4× MIC levels. Data points are the mean from three independent experiments with error bars representing one standard deviation of the mean.

MPTA was bactericidal at 2× and 4× MIC levels, with a time to bactericidal killing of less than 1 hour (Figure 2B). Similar results were observed for BCBP at 2× or 4× MIC levels, with bactericidal killing also observed in 4 hours at 1× MIC and in 24 hours at 0.5× MIC (Figure 2C). Additionally, cells treated with CFAM exhibited killing in 1 hour at 4× MIC and in 4 hours at 2× MIC (Figure 2D).

### Post-antibiotic effect experiments

The PAE of each compound was determined by the equation PAE  =  *T – C*, where *T* is the time required for CFU levels to increase 1-log above the count observed immediately after drug removal, and *C* is the time required for the untreated control CFU to increase 1-log above the count observed after completing the wash procedure used to remove the compounds in the test cultures. The compounds were added to the indicated amounts of 1×, 2×, and 4× MIC after the zero time point. The cells were treated for 60 min followed by thorough washing and transfer to fresh medium. After transfer to fresh medium, aliquots were removed, serially diluted and plated on LB at each time point from 0–8 hours and 24 hours.

In these experiments, *B. subtilis* cultures at 10^6^ CFU/mL were exposed to 1×, 2× or 4× MIC levels of each compound (or a DMSO control) for 60 minutes. Cells were washed to remove the compound and transferred to media lacking the inhibitors, and recovery was assessed by measuring CFUs over time.

### DNA replication rate measurements

*B. subtilis* (PY79) or *E. coli imp4213* were grown in defined S7_50_ minimal media. [28] supplemented with 1% glucose at 37°C to an OD_600_ of 0.3 to 0.4. At indicated time points, 0.5 ml of the culture was removed and added to a pulse-label solution containing 10 µCi/ml [^3^H] thymidine (PerkinElmer, NET027001MC). Cells were pulse-labeled for 5 minutes at 37°C with shaking to allow label incorporation and replication was stopped by the addition of 5 mL of cold 10% TCA (Fisher, BP555-1). Immediately prior to the 20 min time, DMSO (150 µL), MPTA (20 µM), BCBP (16 µM), CFAM (48 µM), or 25 µg/mL nalidixic acid was added. Cell survival was measured at each time point in parallel experiments as previously described [15], [29].

### Protein synthesis rate measurements

*B. subtilis* (PY79) was grown in defined S7_50_ minimal media. [28] supplemented with 1% glucose at 37°C to an OD_600_ of 0.3 to 0.4. At indicated time points, 0.5 ml of the culture was removed and added to a pulse-label solution containing 10 µCi/ml [^3^H] leucine (Moravek, MT672E). Cells were pulse-labeled for 2 minutes at 37°C with shaking to allow label incorporation and replication was stopped by the addition of 5 mL of cold 10% TCA. Immediately prior to the 20 min time, DMSO (150 µL), MPTA (20 µM), BCBP (16 µM), CFAM (48 µM), or nalidixic acid (25 µg/mL) was added.

### RecA-GFP microscopy

Strain LAS40 (*recA*-*mgfp*) was grown in defined S7_50_ minimal media supplemented with 2% glucose at 30°C to an OD_600_ of 0.4. The culture was split, a portion of cells were left untreated while the other portion was treated with MPTA (10 µM), BCBP (8 µM), or CFAM (24 µM) for 5 min. Cells were visualized by fluorescence microscopy [30]–[32] and the percentage of nucleoids with RecA-GFP foci was scored. A small percentage of cells treated with each of the compounds exhibited punctate RecA-GFP localization and/or membrane-associated RecA-GFP, which is not consistent with discrete, nucleoid associated foci. These localizations were scored, but excluded from the calculation.

## Results

### Small molecule inhibitors that block SSB/protein interactions have antibacterial activity

To test for antibacterial activity in SSB PPI inhibitors, colony formation by several diverse bacterial species cultured in the presence of the inhibitors was measured. Of the strains tested, *Bacillus subtilis*, *Neisseria gonorrhoeae*, *N. meningitidis*, *N. lactamica*, and *Listeria monocytogenes* failed to form colonies in the presence of MPTA (50 µM), BCBP (100 µM), or CFAM (100 µM) (Figure 1 and Table 1). BCBP also inhibited growth of *Mycobacterium tuberculosis* and *M. bovis* whereas MPTA and CFAM did not. Several other bacterial species were resistant to the compounds in the plate assay, including *E. coli, M. smegmatis*, *M. avium paratuberculosis*, and *Staphylococcus aureus* (Figure 1 and Table 1). *Saccharomyces cerevisiae*, a model eukaryotic species, was also resistant in the assay. Thus the compounds inhibited the growth of some, but not all, bacterial species tested.

**Table 1:** Sensitivity of diverse species to SSB PPI inhibitors.

Bacterial species	Growth media	No treatment	MPTA (50 µM)	BCBP (100 µM)	CFAM (100 µM)
*E. coli*	LB	+	+	+	+
*E. coli imp-4213*	LB	+	−	−	−
*B. subtilis*	LB/MH	+/+	−/+	−/+	−/+
*N. gonorrhoeae*	GC	+	−	−	−
*N. meningitidis*	GC	+	−	−	−
*N. lactamica*	GC	+	−	−	−
*L. monocytogenes*	LB	+	−	−	−
*S. aureus*	LB/MH	+/+	−/+	−/+	−/+
*M. smegmatis*	MD	+	+	+	+
*M. avium paratuberculosis*	MD	+	+	+	+
*M. tuberculosis*	MD	+	+	−	+
*M. bovis*	MD	+	+	IG	+
*S. cerevisiae*	YPD	+	+	+	+

+ indicates visible growth, – indicates no visible growth, IG – indicates very few colonies compared to the untreated control. LB: Luria broth, MH: Mueller-Hinton, MD: Middlebrook 7H10, GC: GC, YPD: yeast peptone dextrose media.

To test whether the growth media affected inhibitor sensitivity of the strains, the media on which *B. subtilis* (which was sensitive to the compounds in lysogeny broth (LB)) and *S. aureus* (which was resistant to the compounds in Mueller Hinton) were grown was switched in the assay. Consistent with a media dependence to strain sensitivity, *B. subtilis* was more resistant to the compounds when grown on Mueller Hinton whereas *S. aureus* was sensitive when grown on LB (Table 1 and S1). This difference could potentially be explained by sequestration of the compounds by the starch in the Mueller Hinton media, which would reduce their availability to growing cells.

Media sensitivity effects led us to consider whether membrane permeability might also limit cytoplasmic accessibility for the SSB PPI inhibitor insensitivity in some of the strains examined. To test this idea, an *E. coli* strain with increased membrane permeability (*imp4213* [33], [34]) was tested in the growth assay. Unlike wildtype *E. coli*, which was resistant to the compounds, *E. coli imp4213* cells were sensitive to each of the inhibitors (Figure 1 and Table 1). This observation is consistent with the idea that resistance to the SSB PPI inhibitors in some instances may be due to poor cell membrane penetration. Moreover this result strongly supports a cytoplasmic site of action for the compounds, which is expected for inhibitors that disrupt genome integrity.

### Potency and bacteriocidal nature of SSB PPI inhibitors

The potency of each of the PPI inhibitors was assessed in model Gram-positive (*B. subtilis*) and Gram-negative (*E. coli imp4213*) bacterial strains. With *B. subtilis* grown on LB-agar, minimal inhibitory concentration (MIC) values for MPTA, BCBP, and CFAM were 16, 20, and 32 µM, respectively. As a comparison, the MIC for the antibiotic kanamycin measured under the same experimental conditions was 5 µM. With *E. coli imp4213*, the MIC values for MPTA, BCBP, and CFAM were 10, 62, and 36 µM, respectively, whereas the MIC for kanamycin was 3 µM. The full MIC ranges over liquid and solid media are shown in Table S1. Time-kill experiments showed that each of the compounds was bactericidal against *B. subtilis*, killing over 99.9% of cells in 24 hours (Figure 2 A–D).

**Figure 3:**
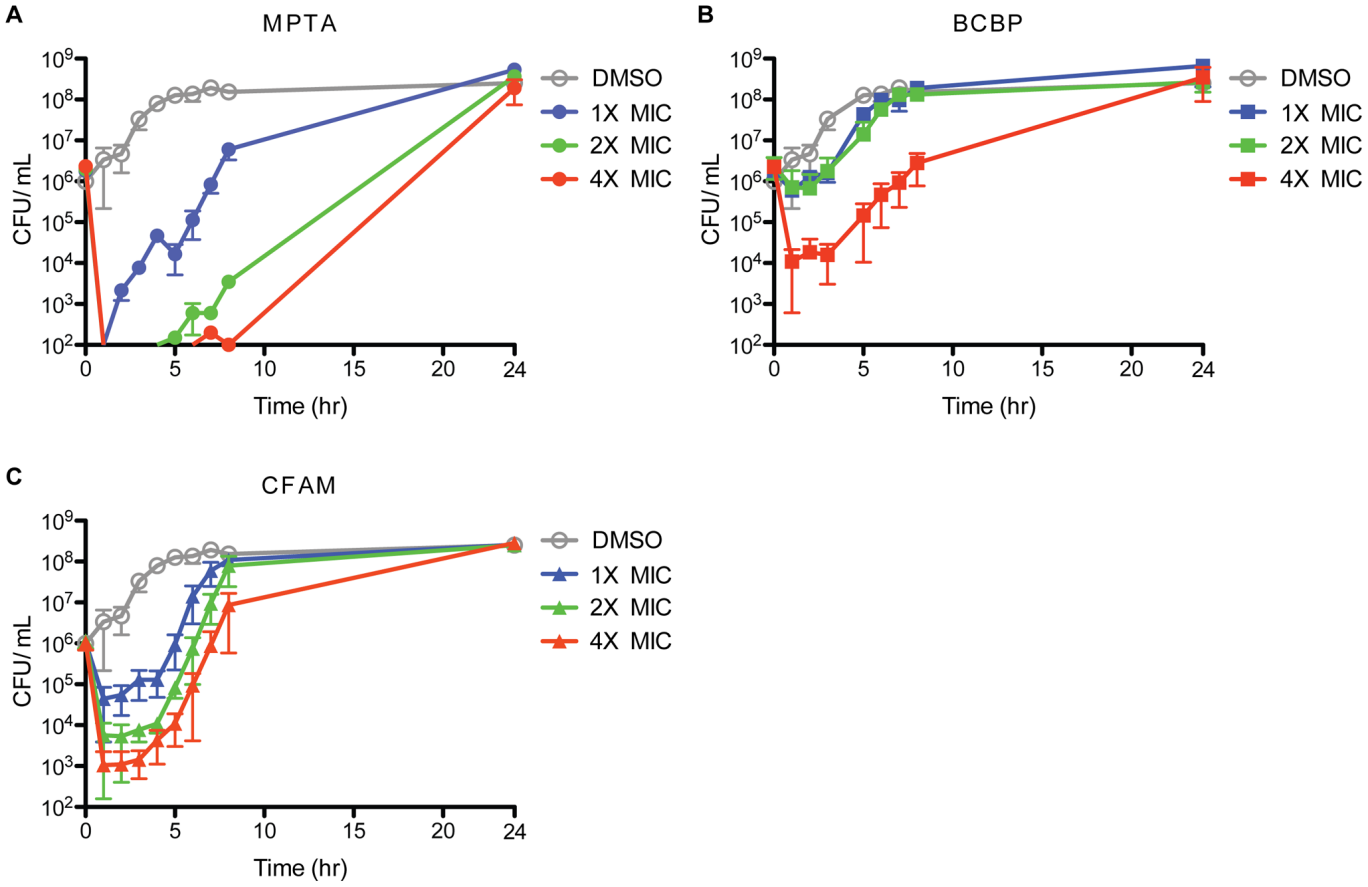
MPTA, BCBP, and CFAM suppress growth after compound removal. Post antibiotic effect of *B. subtilis* PY79 treated with (**A**) MPTA, (**B**) BCBP, or (**B**) CFAM, after 1 hour of treatment with 1×, 2×, or 4× MIC levels. CFUs were measured at time zero (just prior to treatment) and at the times indicated after treatment. Data points are the mean from three independent experiments with error bars representing one standard deviation of the mean.

To assess the kinetics of cell recovery after acute exposure to the compounds, the post-antibiotic effect (PAE) of treatment with each compound was assessed. A long PAE is expected if bacterial growth continues to be suppressed after a short exposure to the antibiotic, which is an important characteristic of a good antibiotic. The PAE for MPTA was concentration dependent whereas the compound concentration did not affect the PAE of either CFAM or BCBP (Figure 3 A–C). These results show that MPTA rapidly and efficiently kills bacteria and that treated cultures need extended recovery time after treatment. In contrast, the PAE for BCBP and CFAM did not change with increasing concentration, indicating that these compounds have a less rapid effect on cells when compared to MPTA.

### Membrane permeability changes lead to SSB PPI inhibitor resistance

We next assessed the mechanism(s) by which bacteria might evolve resistance to the SSB PPI inhibitors. Bacteria can evolve resistance to antibiotics in three primary ways: (1) altering cytoplasmic accessibility, (2) mutating genes encoding the protein(s) targeted by the compound, and (3) producing a protein that degrades the antibiotic. To determine which of these might be pathways for resistance to the SSB PPI inhibitors, we examined the mechanism of resistance of selected spontaneous suppressor mutants of *E. coli imp4213* that allowed growth on the compounds (formed with a frequency of ∼1 mutant per 10^6^ cells). We began by focusing on the possibility that SSB PPI inhibitor resistance could arise from blocking cytoplasmic access for the molecules by suppressing the *imp4213* mutation. The increased membrane permeability of *E. coli imp4213* cells eliminates the ability of the strain to grow on MacConkey's agar due to accumulation of bile salts present in the media to toxic levels in the cell cytoplasm [33], [35]. Suppressors of the *imp4213* mutation are readily detected by their ability to form colonies on MacConkey's agar [33], [35]. Out of 455 *E. coli imp4213* colonies that were able to grow in the presence of the SSB PPI inhibitors, 448 were also able to grow on MacConkey's agar, indicating that resistance was linked to suppression of the *imp4213* hyperpermeability phenotype. Each of the seven compound-resistant colonies that remained sensitive to bile salts exhibited an obvious mucoid phenotype, consistent with changes to capsule [36] that likely exclude the SSB PPI inhibitors but not bile salts. Thus, cytoplasmic exclusion of the compounds appears to be the dominant (and perhaps sole) mode for resistance to the SSB PPI. Interestingly, multiple attempts to generate compound-resistant *B. subtilis* strains failed to produce resistance. This result could indicate that membrane permeability resistance mechanisms to the SSB PPI inhibitors are restricted to a subset of bacterial species and that spontaneous resistance generation by other mechanisms is extremely rare.

### SSB PPI inhibitors disrupt nucleic acid processing in vivo

**Figure 4:**
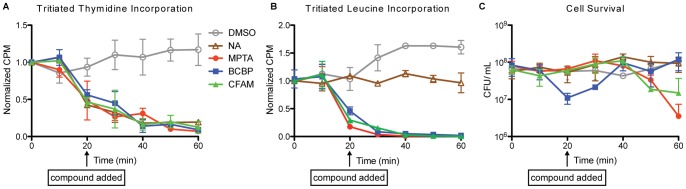
The SSB PPI inhibitors block DNA replication and protein translation in *B. subtilis* prior to cell death. (**A**) Incorporation of [^3^H] thymidine is measured in a pulse experiment in the absence or presence of MPTA (18 µM), BCBP (16 µM), CFAM (48 µM), or Nalidixic acid (NA, 25 µg/mL). Compounds were added to the culture at the 20 min time point. Duplicate samples were measured for each time point and each experiment was repeated in triplicate. All samples were normalized to the time zero reading with the data point as the mean and error bars are one standard deviation from the mean. The line connects the data points for simplicity and does not represent the amount of incorporation. (**B**) Incorporation of [^3^H] Leucine is measured in a pulse experiment in the absence or presence of MPTA (18 µM), BCBP (16 µM), CFAM (48 µM), or Nalidixic acid (NA, 25 µg/mL). Compounds were added to the culture at the 20 min time point. Duplicate samples were measured for each time point and each experiment was repeated in triplicate. All samples were normalized to the time zero reading with the data point as the mean and error bars are one standard deviation from the mean. The line connects the data points for simplicity and does not represent the amount of incorporation. (**C**) CFU/ml of *B. subtilis* under the same conditions as in *A* and *B*. Data points are the mean from all experiments with error bars representing one standard deviation of the mean.

We next examined the effects of the SSB PPI inhibitors on DNA replication, a genome maintenance pathway with predicted sensitivity to blockage of SSB/protein interactions. Pre- and post-PPI-inhibitor treatment replication rates in exponential phase *B. subtilis* and *E. coli imp4213* cultures were measured by pulse labeling aliquots of bacterial cultures with [^3^H] thymidine and quantifying its incorporation into DNA [29]. In control experiments, the addition of DMSO had no effect on [^3^H] thymidine incorporation rates whereas the addition of nalidixic acid, which impairs replication by inhibiting gyrase [37], led to an immediate reduction in the replication rate that was further reduced over the course of the experiment (Figure 4A and S1). The effects of the SSB PPI inhibitors were markedly similar to that of the nalidixic acid positive control; addition of MPTA, BCBP, or CFAM at 2× MIC levels led to an immediate inhibition of replication that was further reduced over time (Figure 4A and S1).

To determine the pathway specificity of the compounds, we also tested whether they affect protein synthesis, which was anticipated to be insensitive to the inhibitors. The effects of the SSB PPI inhibitors on protein translation rates were measured using a [^3^H] leucine pulse labeling approach analogous that used in the DNA replication assay. Adding DMSO and nalidixic acid controls had little to no effect on protein production over the course of the experiment. Unexpectedly, however, the addition of MPTA, BCBP, or CFAM at 2x MIC levels resulted in a rapid inhibition of protein translation (Figure 4B). This unexpected result could indicate that the compounds are generally cytotoxic, with the reduced replication and translation rates stemming from rapid cell death. However, control experiments that measured cell viability (CFUs) in identically treated cultures over the same time course showed that inhibition of DNA replication and protein synthesis preceded cell death caused by the SSB PPI inhibitors (Figure 4C). Thus the effects on translation are more likely to reflect either off-target effects of the compounds on translation or a previously unknown role for SSB/protein interactions in protein synthesis. These possibilities are developed further in the *Discussion* section. Even though the origin of the effect on translation is not clear, the rapid inhibition of replication and translation provides the compounds with a very effective antimicrobial activity against a wide-range of bacterial species.

### SSB PPI inhibitors disrupt RecA focus formation in vivo

We next asked whether the compounds could affect DNA recombination, an additional genome maintenance process that depends on protein interactions with SSB. Activity of the bacterial recombinase, RecA, is limited by its ability to access to ssDNA, which is typically sequestered by SSB. RecA assembly in cells is therefore highly dependent on mediator proteins that bind directly to SSB and modify the SSB/DNA structure to provide RecA access to ssDNA [38], [39]. Assembly of RecA *in vivo* can be quantified by following formation of foci of RecA-GFP fusion proteins [31], [40]. RecA focus formation is linked to DNA replication and to the initiation of recombinational DNA repair of stalled replication forks in *B. subtilis* [31], [40].

RecA-GFP foci were counted in DMSO-control and SSB PPI inhibitor-treated *B. subtilis* cultures. Within 5 minutes, a decrease in the number of foci associated with nucleoids was observed in cells treated with ∼1× MIC levels of the compounds but not in the DMSO controls (Table 2, Figure S2). Foci in cells treated with MPTA decreased by ∼30% relative to the DMSO control, whereas BCBP- and CFAM-treated cells decreased by over 50% and 70% respectively (Table 2). Cell survival was measured over the same time course by incubating the cells with BacLight reagents immediately after compound treatment (Figure S3). In all cases between 77% and 90% of cells survived the treatment (Tables S2 and S3), confirming our earlier results showing that the compounds are not immediately lethal. Thus, as was the case with DNA replication and protein translation, DNA recombination is rapidly inhibited by treatment with the SSB PPI inhibitors. Taken together, these results suggest a model in which multiple nucleic acid processing pathways are blocked simultaneously by the compounds, which leads to large-scale failure of essential processes in vivo.

**Table 2:** RecA-GFP foci are reduced following treatment of cells with small molecules that inhibit interaction with SSB.

Treatment	Nucleoids with foci^a^	Total nucleoids scored	Percentage of nucleoids with foci ±95% CI	P value
Untreated	2250	26,860	8.38±0.331	–
10 µM MPTA	366	6189	5.91±0.588	4.89×10^−11^
8 µM BCBP	286	7595	3.77±0.428	2.36×10^−42^
24 µM CFAM	206	7995	2.58±0.347	4.29×10^−71^

a A small percentage of cells treated with each of the compounds exhibited RecA-GFP localization which was punctate and/or membrane associated, which is not consistent with discrete, nucleoid associated foci. These localizations were scored, but excluded from the calculation shown above. The number of cells with mislocalized RecA-GFP foci are as follows for each compound: MPTA, 28; BCBP, 30; CFAM, 9.

## Discussion

Infections by antibiotic-resistant bacterial strains are a worldwide public health problem. New strategies that identify novel antibacterial targets and lead compounds are needed to help stem this crisis. Here we have examined the question of whether protein interfaces that mediate physical interactions between SSB and its protein partners could provide novel targets for antibacterial development. Nearly every genome maintenance pathway in bacteria depends on interactions with SSB to engage SSB/DNA structures [10], which led to the hypothesis that compounds blocking SSB/protein interactions could simultaneously impair multiple genomic processes, ultimately leading to cell death.

Consistent with this model, we found that small molecule SSB PPI inhibitors impair growth in a wide variety of bacterial species, including disease causing bacteria such as species of *Neisseria* and *L. monocytogenes*. Antibacterial activity required cytoplasmic accessibility, which is consistent with the predicted cytoplasmic mechanism of action of the inhibitors. In agreement with their predicted mechanism, each of the compounds rapidly inhibited DNA replication and recombination initiation *in vivo*. The ability of the compounds to rapidly halt replication may be due to blockage of SSB interactions with the key components of the DNA replication machinery, such as primase or the replicative DNA polymerase DnaE [41]–[43]. Additionally, since the DNA replication restart pathways rely heavily on proteins that interact with SSB to restart stalled replication forks [10], the normal cellular mechanisms aiding in repair of failed replication processes may also be impaired by the SSB PPI inhibitors. In support of this notion, the inhibitors diminish the recombinational repair capacity of the cells, as evidenced by the reduction of RecA foci upon treatment. Recombination initiation could also be directly inhibited by the compounds blocking SSB interaction with the RecO mediator protein, which facilitates RecA loading onto SSB-coated DNA [38], [43], [44].

In addition to their predicted inhibition of DNA replication and recombination, the SSB PPI inhibitors also rapidly disrupted protein synthesis processes. There are several possible explanations for the unexpected ability of the compounds to inhibit translation. The first is that SSB/protein interactions could have important, but previously unrecognized, roles protein translation. Indeed, SSBs from *E. coli* and bacteriophage are known to bind single-stranded RNA in a manner that influences translation [45]–[47] [48], [49]. Moreover, proteomic studies have shown that SSB from both *E. coli* and *B. subtilis* associate with ribosomal subunits [43], [50], although, due to the prevalence of ribosomal proteins as common contaminants in proteome-wide studies, the possibility that these are *bona fide* interactions has not be pursued further. Nonetheless, our observation that translation is blocked by SSB PPI inhibitors could warrant investigations into the roles of SSB in protein synthesis. A second possibility is that the simultaneous impact of the compounds on multiple DNA metabolic processes could indirectly disrupt coupled transcription/translation processes in the bacterial cells, leading to a reduction in protein synthesis. A third possibility is that the compounds could be acting in a non-specific (off-target) manner to block translation.

Antibiotics that directly target genome maintenance, particularly SSB/protein interactions as described here, could provide excellent lead compounds for future broad-spectrum therapeutics. Such inhibitors could act exclusively against bacterial DNA replication, recombination, and repair processes since these pathways are catalyzed by functionally similar but structurally disparate protein complexes in eukaryotes and prokaryotes. To date these distinctions have only been minimally exploited for the development of antibacterial compounds [51]. Unfortunately, the compounds described herein are toxic to human cells in culture (data not shown) and may have off-target effects. Future experiments will be required to modify the structures of the SSB PPI inhibitors to improve their selectivity and their clinical potential.

Taken together, we have shown that direct targeting of PPIs, particularly those that involve SSB, could be an effective strategy for the development of new antibiotic lead compounds. We suggest that similar strategies targeting different essential PPIs or the central genome processes of bacteria could prove important for future development of novel antibacterial compounds that will help alleviate the problem of antibiotic resistance bacterial infections by significantly diversifying our antibacterial arsenal.

## Supporting Information

Figure S1 **The SSB PPI inhibitors block DNA replication and recombination in** ***E. coli imp4213*** **.** Incorporation of [^3^H] thymidine over time is measured in the absence or presence of MPTA (20 µM), BCBP (120 µM), CFAM (72 µM), or Nalidixic acid (NA, 25 µg/mL) added to the culture at the 20 min time point. Duplicate sample were measured for each time point, samples taken every 5 minutes and each experiment was conducted in triplicate. All samples were normalized to the time zero reading, data points are the mean of all three experiments with error bars representing one standard deviation from the mean.
(TIF)

Figure S2 **The SSB PPI inhibitors block recombination in** ***B. subtilis*** **prior to cell death.** Cultures of *B. subtilis* LAS40 (*recA*-*mgfp*) were split; one portion was left untreated and the others were challenged with MPTA (10 µM), BCBP (8 µM), or CFAM (24 µM) for 5 min prior to imaging.
(TIF)

Figure S3 **Treatment of** ***B. subtilis*** **cells with SSB PPI inhibiting small molecules.** *B. subtilis* strains LAS40 (*recA-mgfp*) (left) was grown in defined S7_50_ minimal media supplemented with 2% glucose to an OD_600_ of 0.4. In exponential phase, the cultures were split and a portion of cells were left untreated while the other portion was challenged with MPTA, BCBP, or CFAM as shown for 1 minute. Immediately following challenge, cells were incubated with the BacLight reagents (Invitrogen). Cells were then visualized by microscopy after 5 minutes. Shown are combined images of live (green) and dead (red) cells after treatment with each of the compounds. Treatment with SSB interaction inhibiting small molecules causes only modest killing in wild type cells. Strain PY79 (right) was grown in defined S7_50_ minimal media as in all other experiments. In mid-exponential phase, the culture was split and a portion of the culture was untreated while the other portion was challenged with each of the indicated compounds for 1 minute. Cells were incubated with the BacLight reagents (Invitrogen) immediately following compound challenge. Cells were then visualized by microscopy after 5 minutes.
(TIF)

Table S1 **Minimum inhibitory concentrations and IC50 values.**
(DOCX)

Table S2 **Percent killing of LAS40 (** ***recA-mgfp*** **) cells following challenge with small molecules that inhibit interaction with SSB.**
(DOCX)

Table S3 **Percent killing of LAS508 (PY79) cells following challenged with small molecules that inhibit interaction with SSB.**
(DOCX)
