# Supplementary figures and images for: Protein Interactions in Genome Maintenance as Novel Antibacterial Targets
Source: PLoS One. 2013 Mar 11;8(3):e58765. doi: 10.1371/journal.pone.0058765 (PMC3594151; doi:10.1371/journal.pone.0058765)

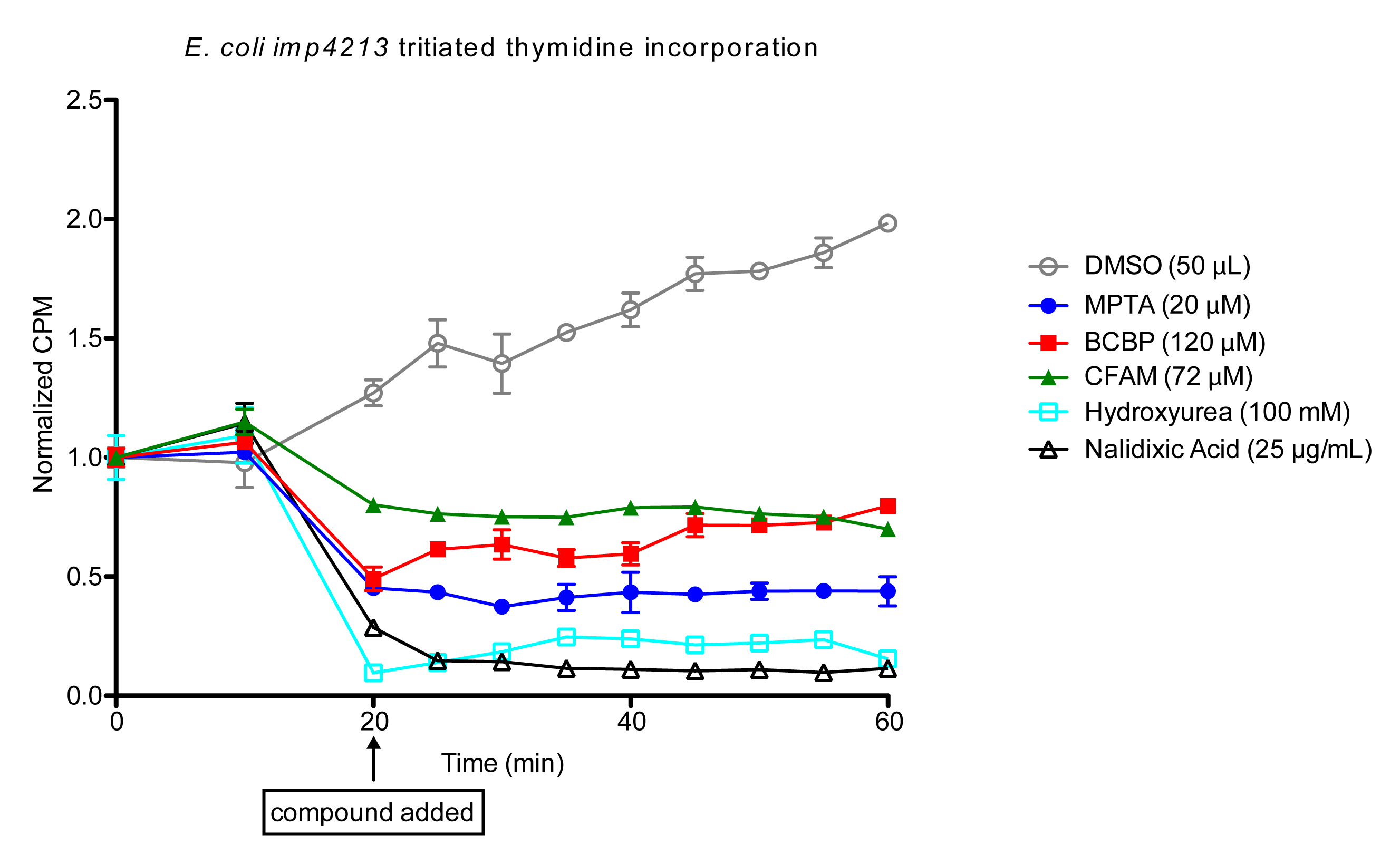

Supplement: Figure S1 — The SSB PPI inhibitors block DNA replication and recombination in E. coli imp4213 . Incorporation of [3H] thymidine over time is measured in the absence or presence of MPTA (20 µM), BCBP (120 µM), CFAM (72 µM), or Nalidixic acid (NA, 25 µg/mL) added to the culture at the 20 min time point. Duplicate sample were measured for each time point, samples taken every 5 minutes and each experiment was conducted in triplicate. All samples were normalized to the time zero reading, data points are the mean of all three experiments with error bars representing one standard deviation from the mean. (TIF) [file pone.0058765.s001.tif]

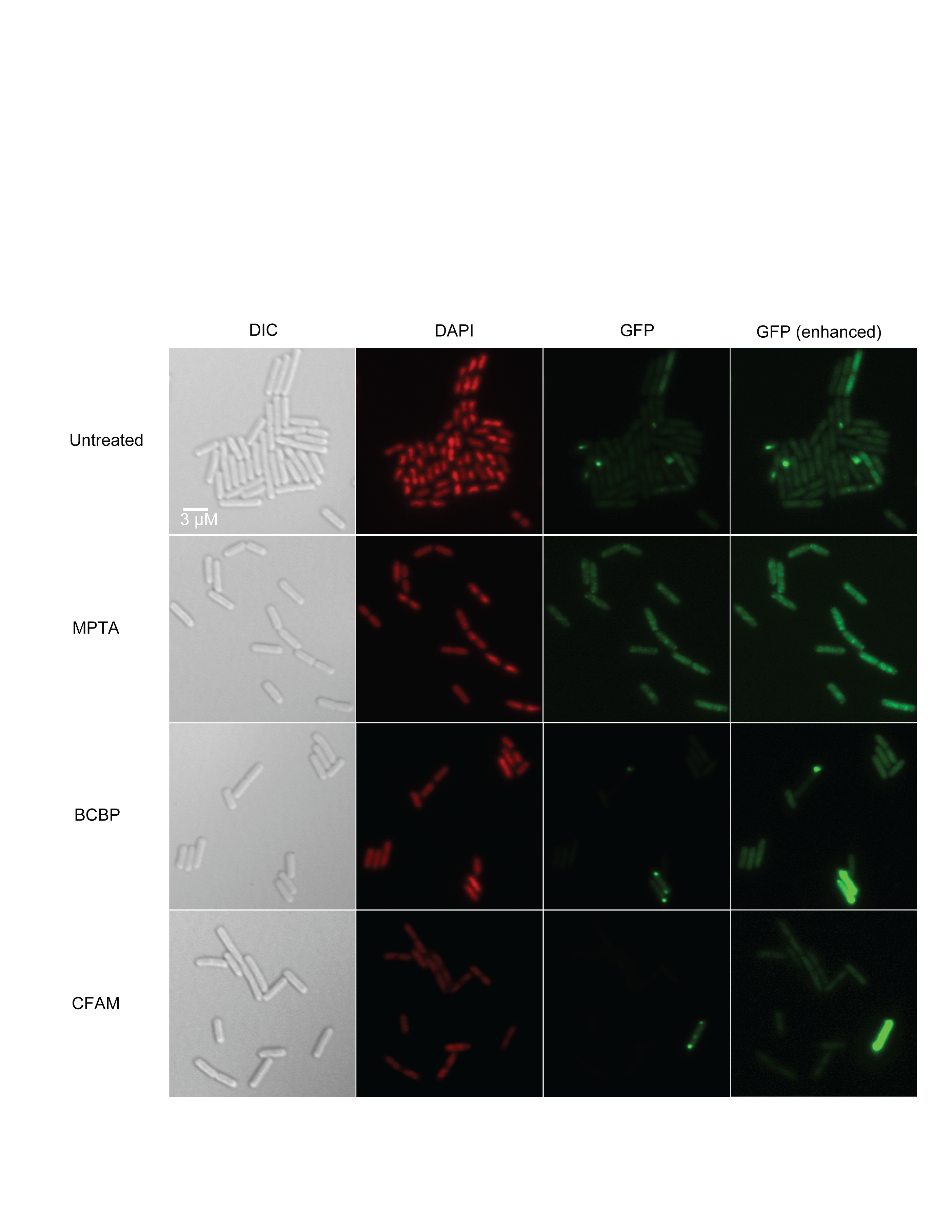

Supplement: Figure S2 — The SSB PPI inhibitors block recombination in B. subtilis prior to cell death. Cultures of B. subtilis LAS40 (recA-mgfp) were split; one portion was left untreated and the others were challenged with MPTA (10 µM), BCBP (8 µM), or CFAM (24 µM) for 5 min prior to imaging. (TIF) [file pone.0058765.s002.tif]

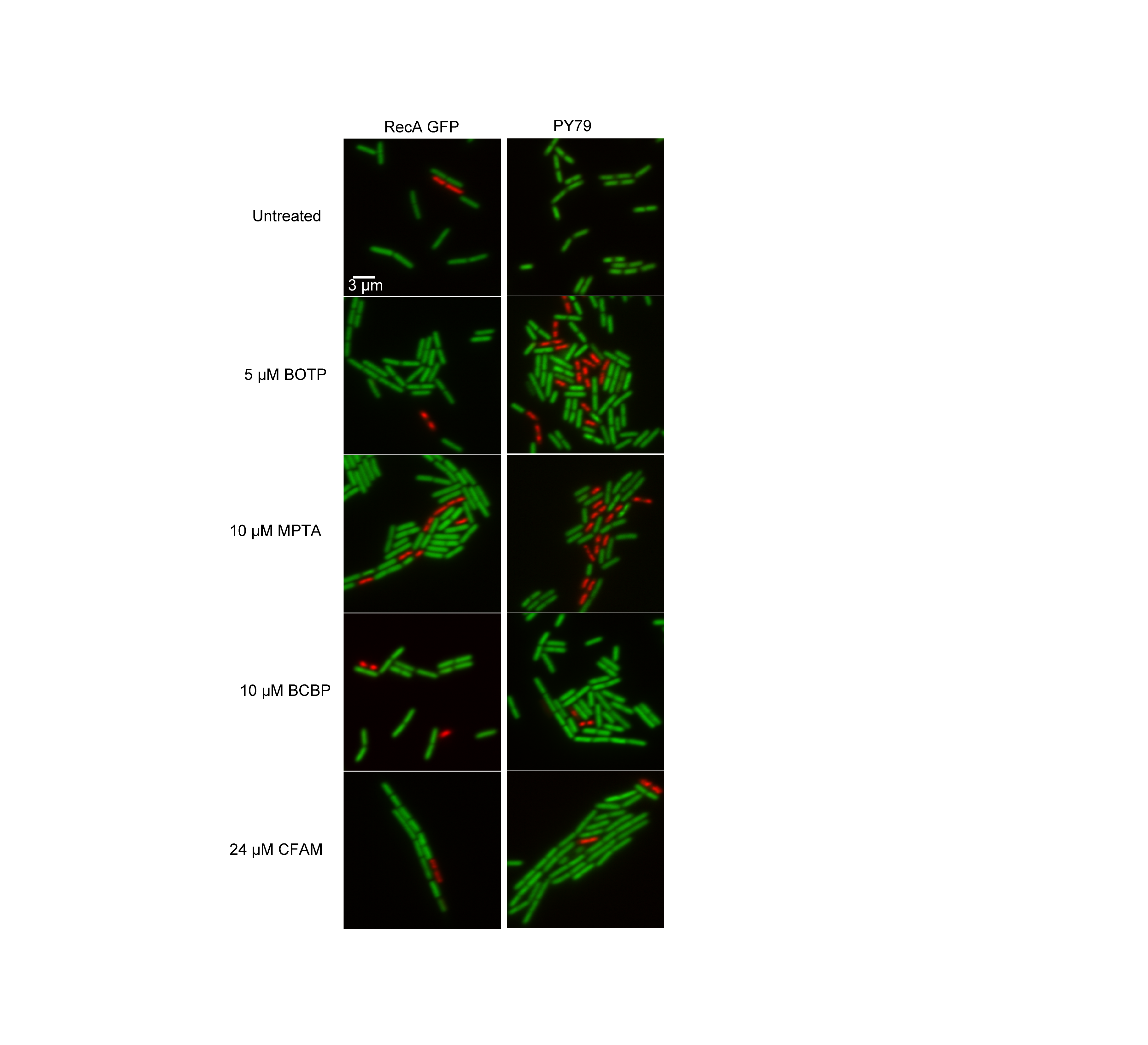

Supplement: Figure S3 — Treatment of B. subtilis cells with SSB PPI inhibiting small molecules. B. subtilis strains LAS40 (recA-mgfp) (left) was grown in defined S750 minimal media supplemented with 2% glucose to an OD600 of 0.4. In exponential phase, the cultures were split and a portion of cells were left untreated while the other portion was challenged with MPTA, BCBP, or CFAM as shown for 1 minute. Immediately following challenge, cells were incubated with the BacLight reagents (Invitrogen). Cells were then visualized by microscopy after 5 minutes. Shown are combined images of live (green) and dead (red) cells after treatment with each of the compounds. Treatment with SSB interaction inhibiting small molecules causes only modest killing in wild type cells. Strain PY79 (right) was grown in defined S750 minimal media as in all other experiments. In mid-exponential phase, the culture was split and a portion of the culture was untreated while the other portion was challenged with each of the indicated compounds for 1 minute. Cells were incubated with the BacLight reagents (Invitrogen) immediately following compound challenge. Cells were then visualized by microscopy after 5 minutes. (TIF) [file pone.0058765.s003.tif]
