# Supplementary material for: Protein Interactions in Genome Maintenance as Novel Antibacterial Targets
Source: PLoS One. 2013 Mar 11;8(3):e58765. doi: 10.1371/journal.pone.0058765 (PMC3594151; doi:10.1371/journal.pone.0058765)
Supplement: Table S1 — Minimum inhibitory concentrations and IC50 values. (DOCX) [file pone.0058765.s004.docx]

Table S1. Minimum inhibitory concentrations and IC50 values

| Strains | MPTA | BCBP | CFAM |
| --- | --- | --- | --- |
| *E. coli imp-4213* | 10 µM | 62 µM | 36 µM |
| *B. subtilis* | 12-16 µM | 8-11 µM | 24-40 µM |
| *B. subtilis* on Mueller Hinton | > 50 µM | > 100 µM | 40-100 µM |
| *S. aureus* on Mueller Hinton | > 50 µM | > 100 µM | > 100 µM |
| *E. coli imp-4213* (IC50) | 11 µM | 6 µM | 30 µM |
| *S. aureus* (IC50) | 29 µM | 5.5 µM | 18 µM |

The ranges listed for *B. subtilis* and *E. coli imp-4213* in the first two rows are the results of MIC tests in both liquid and solid LB. The next row of *B. subtilis* on Mueller Hinton reflects results of growth on solid meida. The MIC values of *S. aureus* on Mueller Hinton are from growth on solid media. The IC50 results are from a 96 well plate assay based on optical density.
