# Supplementary material for: Protein Interactions in Genome Maintenance as Novel Antibacterial Targets
Source: PLoS One. 2013 Mar 11;8(3):e58765. doi: 10.1371/journal.pone.0058765 (PMC3594151; doi:10.1371/journal.pone.0058765)
Supplement: Table S2 — Percent killing of LAS40 ( recA-mgfp ) cells following challenge with small molecules that inhibit interaction with SSB. (DOCX) [file pone.0058765.s005.docx]

Table S2. Percent killing of LAS40 (*recA-mgfp*) cells following challenge with small molecules that inhibit interaction with SSB

| Treatment | Cells alive | Total cells | Percentage of cells living ± 95% CI | P value |
| --- | --- | --- | --- | --- |
| Untreated | 3662 | 3860 | 94.9 ± 0.7 | -- |
| 10 µM MPTA | 1067 | 1384 | 77.1 ± 2.2 | 2.51 × 10^-81^ |
| 8 µM BCBP | 2087 | 2323 | 89.9 ± 1.2 | 3.25 × 10^-14^ |
| 24 µM CFAM | 1270 | 1616 | 78.6 ± 2.0 | 1.13 × 10^-75^ |

Strain LAS40 (*recA-mgfp*) was grown in defined S7_50_ minimal media supplemented with 2% glucose to an OD_600_ of 0.4. In exponential phase, the culture was split and a portion of cells were left untreated while the other portion was challenged with MPTA, BCBP, or CFAM as indicated for 1 minute. Immediately following challenge, cells were incubated with the BacLight reagents (Invitrogen). The cells were then visualized by microscopy on 1% agarose pads after 5 minutes, and the number of dead cells was scored relative to the number of live cells in each culture. The 95% confidence interval is reported and the p-value indicating statistical significance.
