# Supplementary material for: Protein Interactions in Genome Maintenance as Novel Antibacterial Targets
Source: PLoS One. 2013 Mar 11;8(3):e58765. doi: 10.1371/journal.pone.0058765 (PMC3594151; doi:10.1371/journal.pone.0058765)
Supplement: Table S3 — Percent killing of LAS508 (PY79) cells following challenged with small molecules that inhibit interaction with SSB. (DOCX) [file pone.0058765.s006.docx]

Table S3. Percent killing of LAS508 (PY79) cells following challenged with small molecules that inhibit interaction with SSB

| Treatment | Cells alive | Total cells | Percentage of cells living ± 95% CI | P value |
| --- | --- | --- | --- | --- |
| Untreated | 8086 | 8250 | 98.0 ± 0.3 | -- |
| 10 µM MPTA | 1834 | 2404 | 76.3 ± 1.7 | 4.79 × 10^-300^ |
| 8 µM BCBP | 2670 | 2731 | 97.8 ± 0.5 | 0.216 |
| 24 µM CFAM | 3208 | 3652 | 87.8 ± 1.1 | 8.92 × 10^-120^ |

Strain LAS508 (PY79) was grown in defined S7_50_ minimal media supplemented with 2% glucose to an OD_600_ of 0.4. During exponential phase, the culture was split and a portion of culture was untreated while the other portion was challenged with MPTA, BCBP, or CFAM as indicated for 1 minute. Immediately following challenge, cells were incubated with the BacLight reagents (Invitrogen). The cells were then visualized by microscopy after 5 minutes, and the number of dead cells were scored relative to the number of live cells in each culture. The 95% confidence interval (CI) is indicated as well as the p-value for statistical significance.
